# Ulceration of the Stomach of a Horse, and Chronic Glanders of a Mule

**Published:** 1897-11

**Authors:** W. E. A. Wyman

**Affiliations:** Clemson Agricultural and Mechanical College, South Carolina


### ULCERATION OF THE STOMACH OF A HORSE, AND CHRONIC GLANDERS OF A MULE.

By W. E. A. WYMAN, V.S.,
CLEMSON AGRICULTURAL AND MECHANICAL COLLEGE, SOUTH CAROLINA.

RECENTLY a horse was brought to the free clinic, supposed to be suffering with colic. The owner informed the writer that the horse had colic frequently, that his bowels were at times "loose," then again constipated. Whenever drinking water, especially on an empty stomach, the horse would drink a little and then with an anxious expression, look at the flank. When presented for treatment, the horse was sick for two hours, and had received no medical attention. Examination of the animal revealed the following: General emaciation; mucous membranes cyanotic; intense dyspnœa; pulse hardly perceptible, and above 120; epigastric region very full; animal covered with cold, clammy sweat; kneels down occasionally for a second or two frequent belching and vomiting of a sour-smelling mass streaked with blood. Diagnosis of rupture of the stomach was made. The animal died about fifteen minutes after being brought to the clinic. Post-mortem examination made within twenty minutes after death showed a rent of about one inch near the middle of the great curvature of the stomach. On opening the stomach, an ulcerated condition extending across the whole stomach in a zigzag fashion, following apparently the division between the œsophageal and pyloric portion of that organ, was visible. The ulcer was covered with a black adhering crust, reminding one forcibly of those seen in chronic hog-cholera. Another ulcer, about one and one-half inches in length and one-half inch wide, was found on the pyloric sphincter muscle. Throughout the whole intestinal tract, especially in the small intestines, strictures varying in length from one to five inches were noticeable.

While examining a number of horses and mules for the State—supposed to be suffering with glanders—a mule with chronic glanders, ulceration and perforation of the nasal mucous membrane and septum nasi, was met with. Post-mortem showed lungs with tubercles in all stages.

---

Goat's milk is not as digestible as cow's milk, and forms in the stomach a much denser and harder curd.

Moving microbes are now photographed by the micromotoscope, and their actions and progress studied under conditions that will shed much light on true pathology.

Antiseptic powder: iodoform, salol, bismuth subnitrate, charcoal, cinchona, benzoin, equal parts.

Antiseptic varnish: powdered lac, 900 grains; balsam of tolu, 75 grains; thymol, 22 grains; alcohol, 750 grains; ordinary ether, 1500 grains; filter.

The *Bacteriological Review* quotes Dr. Wilcox as saying of strophanthus that it is the choice of drugs in heart difficulties. 1. In all cases in which we wish to establish compensation. 2. In all cases of arterial degeneration in which a remedy which causes more energetic cardiac contraction is required. 3. In all cases of cardiac diuresis where diuresis is necessary. 4. In all cases of weak or irritable heart. 5. In all cases of cardiac disease of very young or of old age.

